# Characterizing the frequency of clinical events and assessing biomarkers in propionic acidemia: a natural history study

**DOI:** 10.1186/s13023-026-04251-3

**Published:** 2026-03-09

**Authors:** Bernd C. Schwahn, Gerard T. Berry, Hilary J. Vernon, Hong Li, J. Lawrence Merritt II, Manuel Schiff, Brigitte Chabrol, Javier De las Heras, Jerry Vockley, Chung Lee, Dwight D. Koeberl, Barbara K. Burton, Stephanie Grunewald, George A. Diaz, Can Ficicioglu, Thomas Morgan, Junxiang Luo, Husain Attarwala, Min Liang, Sue Perera, Vanja Sikirica

**Affiliations:** 1 Manchester Centre for Genomic Medicine, St Mary’s Hospital, Manchester University NHS Foundation Trust, Health Innovation Manchester, Manchester, UK; 2 Division of Evolution & Genomic Sciences, School of Biological Sciences, Faculty of Biology, Medicine and Health, University of Manchester, Manchester, UK; 3 Division of Genetics and Genomics Boston Children’s Hospital and Harvard Medical School, Boston, MA USA; 4 Department of Genetic Medicine, Johns Hopkins University, Baltimore, Maryland, USA; 5 Department of Human Genetics, School of Medicine, Emory University, Atlanta, GA USA; 6 Division of Genetic Medicine, Department of Pediatrics, University of Washington and Seattle Children’s Hospital, Seattle, WA USA; 7 Reference Center for Inborn Errors of Metabolism, Necker University Hospital, APHP and University of Paris Cité, Filière G2m, MetabERN, Paris, France; 8 INSERM UMRS_1163, Institut Imagine, Paris, France; 9 Reference Center for Inherited Metabolic Disorders, Assistance Publique Hôpitaux de Marseille, Centre Hospitalier Universitaire de La Timone Enfants, Filière G2m, MetabERN, Marseille, France; 10 Hereditary Metabolic Diseases Unit at Hospital Universitario Cruces (CIBER-ER), Bio-Bizkaia Health Research Institute, University of the Basque Country (UPV/EHU), Barakaldo, Spain; 11 Division Genetic and Genomic Medicine, Department of Pediatrics, University of Pittsburgh, Pittsburgh, PA USA; 12 Department of Pediatrics, Division of Medical Genetics, Stanford University, Stanford, CA USA; 13 Division of Medical Genetics, Department of Pediatrics, Duke University School of Medicine, Durham, NC USA; 14 Ann & Robert H. Lurie Children’s Hospital and Northwestern University Feinberg School of Medicine, Chicago, IL USA; 15 Great Ormond Street Hospital for Children and Institute of Child Health, NIHR Biomedical Research Center, London, UK; 16 Icahn School of Medicine at Mount Sinai, New York, NY USA; 17 Children Hospital of Philadelphia, Philadelphia, PA USA; 18 Vanderbilt University School of Medicine, Nashville, TN USA; 19 West Virginia University School of Medicine, Morgantown, WV USA; 20 Moderna, Inc., Cambridge, MA USA

**Keywords:** Propionic acidemia, Propionyl-CoA carboxylase deficiency, Metabolic decomposition events, Biomarkers, Clinical events, 3-HP, 2-MC, C3

## Abstract

**Background:**

Propionic acidemia (PA) is a rare disease resulting in toxic accumulation of metabolites that cause metabolic decompensation events (MDEs). Natural history characterization of PA is limited partly due to its rarity and complexity. This study examined clinical events in participants with PA and their correlation with biomarkers to better understand the disease.

**Methods:**

Participants diagnosed with PA (early- versus late-onset) were evaluated every 6 months from baseline to month 36. Outcome measures included annualized MDEs and changes in 3-hydroxypropionic acid (3-HP), 2-methylcitrate (2-MC), and propionylcarnitine (C3) concentrations. Blood samples were collected at each visit and during MDEs for plasma biomarker analyses. Relative risk (RR) was calculated based on MDE rates and biomarker levels.

**Results:**

Most participants (n/*N* = 49/50, 98.0%) reported MDEs. Annualized MDE rates were highest in participants aged 0 to ≤ 1 and > 1 to ≤ 2 versus > 12 to ≤ 18 years (2.6 and 2.4, respectively, vs 0.2) and for early-onset versus late-onset PA (1.2 vs 0.3). A 50% decrease in plasma biomarkers was associated with a statistically significant reduction in RR of MDEs for 3-HP (RR: 0.7, 95% CI: 0.6–0.9; *p* < 0.01) and a substantial reduction for C3 (RR: 0.6, 95% CI: 0.4–0.9; *p* = 0.017); no effect was observed for 2-MC.

**Conclusions:**

MDE rates were higher in the youngest participants (0 to ≤ 2 years) versus participants aged > 12 years, and with early- versus late-onset PA. Lower concentrations of 3-HP and C3 were associated with a reduced MDE risk. These findings may guide biomarker or clinical endpoints in future treatment trials.

**Supplementary Information:**

The online version contains supplementary material available at 10.1186/s13023-026-04251-3.

## Background

Propionic acidemia (PA) is a life-threatening metabolic disorder caused by a deficiency of propionyl-coenzyme A (CoA) carboxylase, an enzyme composed of alpha and beta subunits encoded by the *PCCA* (HGNC:8653) and *PCCB* (HGNC:8654) genes [1, 2]. Mutations in the *PCC* genes result in deficient propionate catabolism and thus deficient catabolism of amino acids (valine, methionine, threonine, and isoleucine), cholesterol side chains, and odd-chain fatty acids [1, 3, 4]. Deficient propionyl-CoA carboxylase activity leads to accumulation of toxic metabolites, including 3-hydroxypropionic acid (3-HP), 2-methlycitrate (2-MC), and propionylcarnitine (C3), all of which are considered biomarkers of disease progression and severity, though further investigation of their clinical utility is needed [1, 3–5].

PA is considered an ultra-rare disorder, with an estimated incidence of approximately 1 in 50,000–250,000 newborns and wide demographic variability [2, 3]. Neonatal PA is the most common clinical form of PA (approximately 70–90% of cases) [4], with participants experiencing a variety of symptoms, including lethargy, poor feeding, vomiting, and hypotonia [5–7]. PA can also occur any time after the neonatal period, when more varied signs and symptoms may be displayed, including developmental delay, intellectual disability, failure to thrive, and cardiac and/or kidney damage [1, 7]. Without prompt identification and appropriate management, participants can develop metabolic decompensation events (MDEs), where the body experiences a rapid decline in metabolic function whilst biochemical parameters deteriorate, leading to metabolic acidosis and/or hyperammonemia and serious biochemical instability. MDEs can lead to progressive clinical deterioration, potentially resulting in encephalopathy, cardiorespiratory failure, coma, and death [1, 7, 8]. Regardless of disease onset, presence of MDEs, or other clinical factors, participants with PA experience a high symptomatic burden, require multiple in- and outpatients visits, and may develop complications across multiple organ systems [9].

While newborn screening (NBS) is important for early disease identification and initiation of treatment [6], to date there are no approved disease modifying therapies for PA. Current treatment paradigms for PA predominantly focus on avoiding disease complications such as MDEs, largely through limiting dietary protein, use of antibiotics to diminish propionyl-CoA–producing gut bacteria, and levocarnitine supplementation to convert propionyl-CoA into nontoxic propionylcarnitine. If MDEs continue to persist, liver transplantation may be recommended to provide enzymatic activity reducing MDE frequency and severity, which can lower long-term disease burden [7, 10]. Due to the rarity of PA, and its heterogeneity [11], characterization of the natural history of the disease is challenging, but vital, to establishing future treatment decisions and developing new therapeutic strategies and trial protocols. The purpose of this study was to collect clinical and biomarker data from a population of participants with PA (both early- and late-onset) to better delineate the natural history of the disease, characterize MDEs and other clinical outcomes over time, and determine whether any potential correlations exist between clinical events and biomarkers associated with PA.

## Materials and methods

### Study design

This was an ambispective (use of preexisting data to allow for prospective monitoring), longitudinal, natural history study of participants with PA across 25 sites in North America and Europe. The overall study investigated the natural history of both PA and methylmalonic acidemia (NCT03484767); the MMA portion of the study is being published separately.

**Fig. 1:**
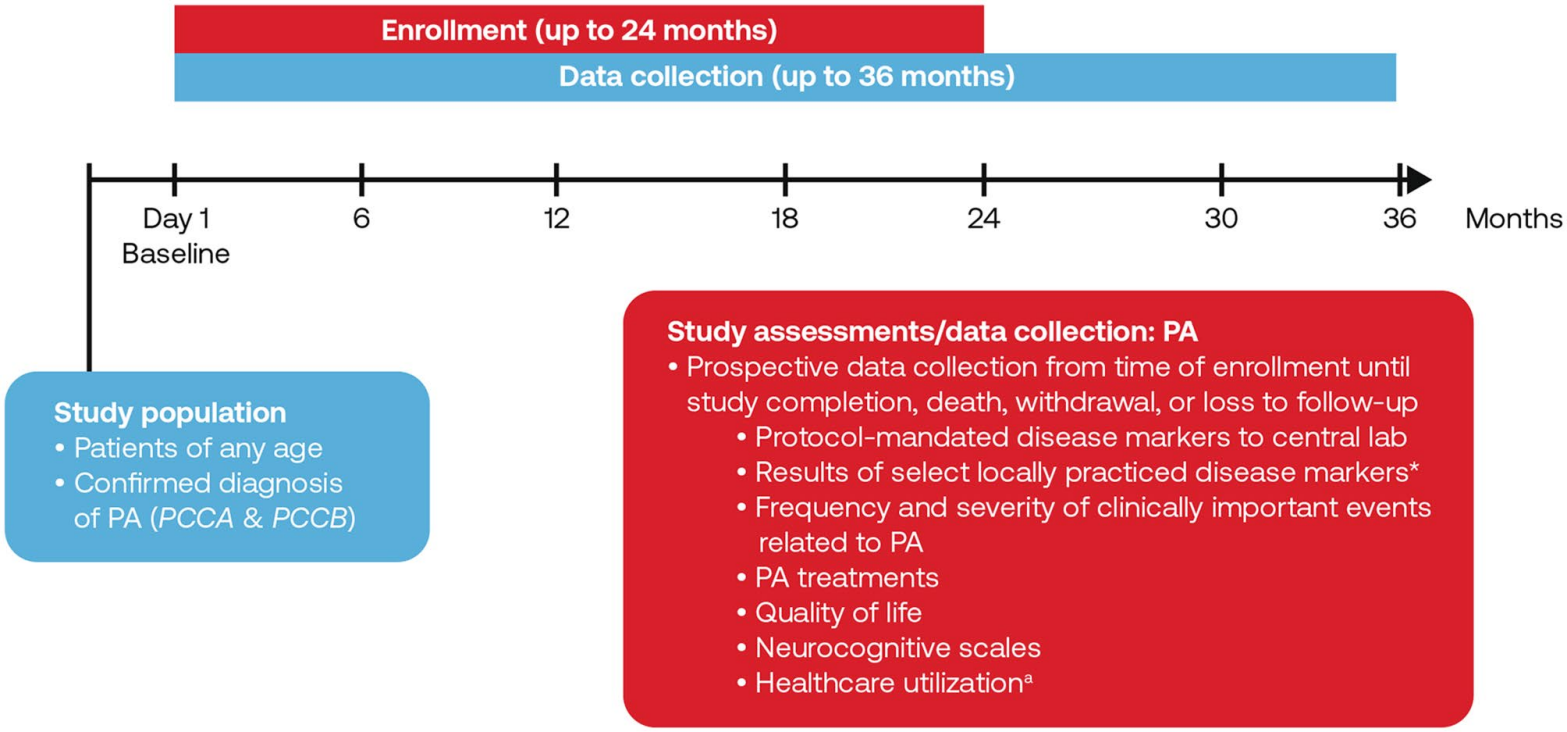
Study design. PA, propionic acidemia; *PCCA*, propionyl-CoA carboxylase alpha subunit; *PCCB*, propionyl-CoA carboxylase beta subunit. *retrospective data collection of above noted assessments

In brief, the study duration (from first participant enrolled to last participant completing the study) was 36 months, with sites enrolling eligible participants with PA (*PCCA* or *PCCB*) during a period of 24 months. Each participant participated in the study for a targeted minimum of 12 months to a maximum of 36 months (Fig. 1). Prospective data collection occurred from the time of enrollment.

### Study population

Participants of any age were required to meet all the following inclusion criteria: confirmed diagnosis of PA based on the following criteria: elevated plasma//Dried Blood Spot (DBS) or urine 2-MC and/or 3-HP levels; elevated plasma/serum/DBS C3; confirmation of biallelic pathogenic variants in the *PCCA* or *PCCB* genes by molecular genetic testing.

### Study endpoints

The primary outcome measures were:

1. The frequency of clinical events from baseline to 12 months. Clinical events included MDEs; digestive issues and abnormal feeding; neurological, renal, cardiac, hematologic or ophthalmological complications; infections; hearing loss.
2. Change in plasma 2-MC, C3, and 3-HP concentrations from baseline to 12 months.

Secondary outcome measures in this study were:

1. Frequency of clinical events from baseline to 6, 18, 24, 30, and 36 months.
2. Change in plasma 2-MC, C3, and 3-HP concentrations from baseline to 6, 18, 24, 30, and 36 months.
3. Correlation between disease markers for PA (2-MC, C3, and 3-HP) and clinical events (MDEs and PA complications).

For all outcomes, participants were stratified by age at enrollment ( > 1 m to ≤ 2 years, > 2 years to ≤ 12 years, > 12 years to ≤ 18 years, > 18 years) and disease onset (early-onset versus late-onset). For analyses by disease onset, early onset was defined as participants who experienced their first MDE within three months of age, and late onset was defined as those who experienced their first MDE after three months of age.

### Analysis of MDEs and biomarkers

MDEs were defined as the presence of any of the following signs/symptoms or laboratory findings, based on the clinical judgement of the investigator: hyperammonemia, and/or metabolic acidosis, with the presence of at least one of the following: vomiting, food refusal or intolerance (different from the participant’s usual pattern), lethargy/decreased activity, impaired consciousness/decreased cognition, or parental concern/other. For annualized MDE rate analyses, participants were stratified by the following age groups: > 1 month to ≤ 2 years, > 2 years to ≤ 4 years, > 4 years to ≤ 12 years, > 12 years to ≤ 18 years, and > 18 years. For subsequent analyses of the signs and symptoms of MDEs and common MDE triggers, the age groups > 2 years to ≤ 4 years and > 4 years to ≤ 12 years were merged into one group ( > 2 years to ≤ 12 years) due to fewer MDEs reported for participants within these age groups. Data were only reported if available for at least five participants at specific time points. To identify the most common signs and symptoms of MDEs and MDE triggers displayed by participants, a threshold of 25% was established.

### Statistical analyses

Due to the rarity of PA, a sample size of up to 60 participants was justified using statistical estimates derived from 300 simulated distributions for urine 3-HP concentrations (μmol/L). Missing data were imputed for date of birth and diagnosis date if the ‘day’ was missing; 0.5 months were added to the provided month and year. The proportion of participants with an MDE was calculated based on the number of participants who attended each visit, not the total population. Summary statistics were analyzed to evaluate the primary and secondary outcome measures. All analyses were performed in the full analysis set, which contained all enrolled participants who signed informed consent and met eligibility criteria. Primary outcome analyses included measuring the proportion of participants with clinical events and number of clinical events in PA, including the proportion of participants experiencing symptoms of an acute decompensation event and number of times the event occurred. Changes in PA disease biomarkers from baseline to month 12 were summarized using continuous variable descriptive statistics. Secondary outcome analyses included measuring the proportion of participants with clinical events and number of clinical events experienced overall and from baseline to months 6, 18, 24, 30, and 36 visits.

In addition to the binomial regression mode, additional analyses were undertaken to assess the correlation between MDEs and 3-HP, a key biomarker for PA [1]. This analysis was conducted to support potential dose selection modelling for mRNA independently, with 3-HP modelled to determine if it could show a correlation since previous literature reviews indicated that 3-HP might be associated with MDE outcomes, potentially establishing it as a surrogate biomarker for clinical outcomes of interest (e.g. MDE) for regulatory purposes. A generalized additive mixed modeling (GAMM) framework was employed, implemented using the R package ‘pammtools’ [12]. Model fitting was performed through restricted maximum likelihood estimation. The onset of MDEs was modeled using a hazard function influenced by three primary factors: (1) the concentration of 3-HP, (2) a smoothed term capturing the number of days since the end of the previous MDE (or the start of the observation window or birth), and (3) a smoothed random effect term reflecting individual participant variability. To assess the model’s suitability for its intended purpose, visual predictive checks were performed. Two primary outcomes were evaluated: the annualized MDE rate and the proportion of participants with annualized MDE rates of at least one. These checks were conducted to compare observed data with model predictions and validate the model’s predictive performance.

All computations and generation of tables, listings, and data for figures were performed using SAS^®^ version 9.4 (SAS Institute, Cary, NC, USA) and R Statistical Software version 4.1.2 (R Core Team 2021), Vienna, Austria.

## Results

### Baseline demographics and disease characteristics

A total of 50 participants with PA consented to participate in the study, all of whom were included. Of these 50 participants, 45 (90.0%) completed the study, while five (10.0%) participants discontinued due to loss of follow-up (*n* = 2, 4.0%) or other reasons (*n* = 3, 6.0%), which included two instances of a site PI leaving with no replacement and one participant transferring care to another center. The median (range) age of participants at enrollment was 9.2 (0.2, 36.0) years and at disease onset was 0.5 (0.0, 146.0) months. Fifty-two percent of participants were female and most participants (70.0%) were non-Hispanic or non-Latino. Most participants had early-onset PA (*n* = 39, 78.0%) compared with late-onset PA (*n* = 11, 22.0%). More enrolled participants with PA had variants in the *PCCB* gene (n/*N* = 28/50, 56%) than the *PCCA* gene (n/*N* = 22/50, 44%) (Table 1).

**Table 1:** Baseline demographics and disease characteristics of study patients, overall and by disease onset

Parameter	Total (*N* = 50)
Median age (range), years	9.2 (0.2–35.6)
Age groups, n (%)	
> 1 month to ≤ 2 years	5 (10.0)
> 2 years to ≤ 12 years	25 (50.0)
> 12 years to ≤ 18 years	9 (18.0)
> 18 years	11 (22.0)
Sex, n (%)	
Male	24 (48.0)
Female	26 (52.0)
Weight, mean (SD), kg	33.9 (19.6)
Ethnicity, n (%)	
Hispanic or Latino	3 (6.0)
Not Hispanic or Latino	35 (70.0)
Unknown/missing^a^	12 (24.0)
Race, n (%)	
Asian	9 (18.0)
Black or African American	6 (12.0)
Native Hawaiian/Other Pacific Islander	0
White	21 (42.0)
Not Applicable	1 (2.0)
Other	4 (8.0)
Unknown/missing/not reported^a^	9 (18.0)
Disease identification, n (%)	
Newborn screening	8 (16.0)
Clinical presentation	39 (78.0)
Family history	3 (6.0)

Three patients had missing phenotype data and are not reported separately here. ^a^Race and/or ethnicity were not reported for some patients because of a discrepancy in categorization between US and European study sites. N, number of patients who signed informed consent and met protocol eligible criteria; n, number of patients who have non-missing data at corresponded data category and timepoint; PA, propionic acidemia

### Disease complications and symptoms at baseline

Nearly all (n/*N* = 49/50, 98.0%) participants enrolled in the study had a history of disease complications and symptoms at baseline (Table 2). The most prominent disease comorbidities observed across all age groups were seizures (n/*N* = 24/49, 49.0%), pancreatitis (n/*N* = 10/49, 20.4%), and renal tubular acidosis (n/*N* = 8/49, 16.3%). Overall, 7/49 participants (14.3%) had cardiomyopathy and 5/49 (10.2%) had a prolonged QT. Participants aged > 1 month to ≤ 2 years (*n* = 5) commonly experienced seizures (n/*N* = 2/5, 40.0%) and renal tubular acidosis (n/*N* = 1/5, 20.0%). Participants aged > 2 years to ≤ 12 years (*n* = 25) commonly experienced seizures (n/*N* = 12/25, 48.0%) and pancreatitis (n/*N* = 7/25, 28.0%). Participants aged > 12 years to ≤ 18 years (*n* = 9) predominantly experienced seizures (n/*N* = 5/8, 62.5%). Participants > 18 years (*n* = 11) most commonly experienced seizures (n/*N* = 5/11, 45.5%) and hearing loss (n/*N* = 4/11, 36.4%).

**Table 2:** Disease complications at baseline – patients with PA by age cohort and disease onset

Parameters		Age cohort				Disease onset	
	Total (*N* = 50)	> 1 month to ≤ 2 years (*n* = 5)	> 2 to ≤ 12 years (*n* = 25)	> 12 to ≤ 18 years (*n* = 9)	> 18 years (*n* = 11)	Early onset (*n* = 39)	Late onset (*n* = 11)
Patients with history of disease complications, N1%)	49 (98.0)	5 (100.0)	25 (100.0)	8 (88.9)	11 (100.0)	39 (100.0)	10 (90.9)
Ongoing or past disease complications, n/N1%)							
Seizures	24/50 (49.0)	2/5 (40.0)	12/25 (48.0)	5/8 (62.5)	5/11 (45.5)	21/39 (53.8)	3/10 (30.0)
Pancreatitis	10/49 (20.4)	0	7/25 (28.0)	0	3/11 (27.3)	9/39 (23.1)	1/10 (10.0)
Renal tubular acidosis	8/49 (16.3)	1/5 (20.0)	4/25 (16.0)	2/8 (25.0)	1/11 (9.1)	8/39 (20.5)	0
Hearing Loss	7/49 (14.3)	0	3/25 (12.0)	0	4/11 (36.4)	5/39 (12.8)	2/10 (20.0)
Cardiomyopathy	7/49 (14.3)	0	3/25 (12.0)	1/8 (12.5)	3/11 (27.3)	6/39 (15.4)	1/10 (10.0)
Metabolic stroke	5/49 (10.4)	0	4/25 (16.0)	0	1/11 (9.1)	4/39 (10.3)	1/10 (11.1)
Optic nerve atrophy	5/49 (10.4)	0	2/25 (8.0)	1/8 (12.5)	2/11 (18.2)	3/39 (7.7)	2/10 (20.0)
Renal failure	3/49 (6.1)	0	0	0	3/11 (27.3)	3/39 (7.7)	0

N1, number of patients who had study disease complications at baseline; n, number of patients with ongoing/past disease complications

All participants with early-onset PA (n/*N* = 39/39, 100.0%) had a history of disease complications and symptoms at baseline, with the most reported complications being seizures (n/*N* = 21/39, 53.8%), pancreatitis (n/*N* = 9/39, 23.1%), and renal tubular acidosis (n/*N* = 8/39, 20.5%). Nearly all participants with late-onset PA (n/*N* = 10/11, 90.9%) had a history of disease complications and symptoms at baseline, with commonly reported complications of seizures (n/*N* = 3/10, 30.0%), optic nerve atrophy (n/*N* = 2/10, 20.0%), and hearing loss (n/*N* = 2/10, 20.0%). A higher proportion of participants with early-onset PA had cardiomyopathy, but a similar proportion had prolonged QT relative to participants with late-onset PA (15.4% (*n* = 6) vs 10% (*n* = 1) and 10.3% (*n* = 4) vs 10.0% (*n* = 1), respectively).

### Analysis of MDEs

**Fig. 2:**
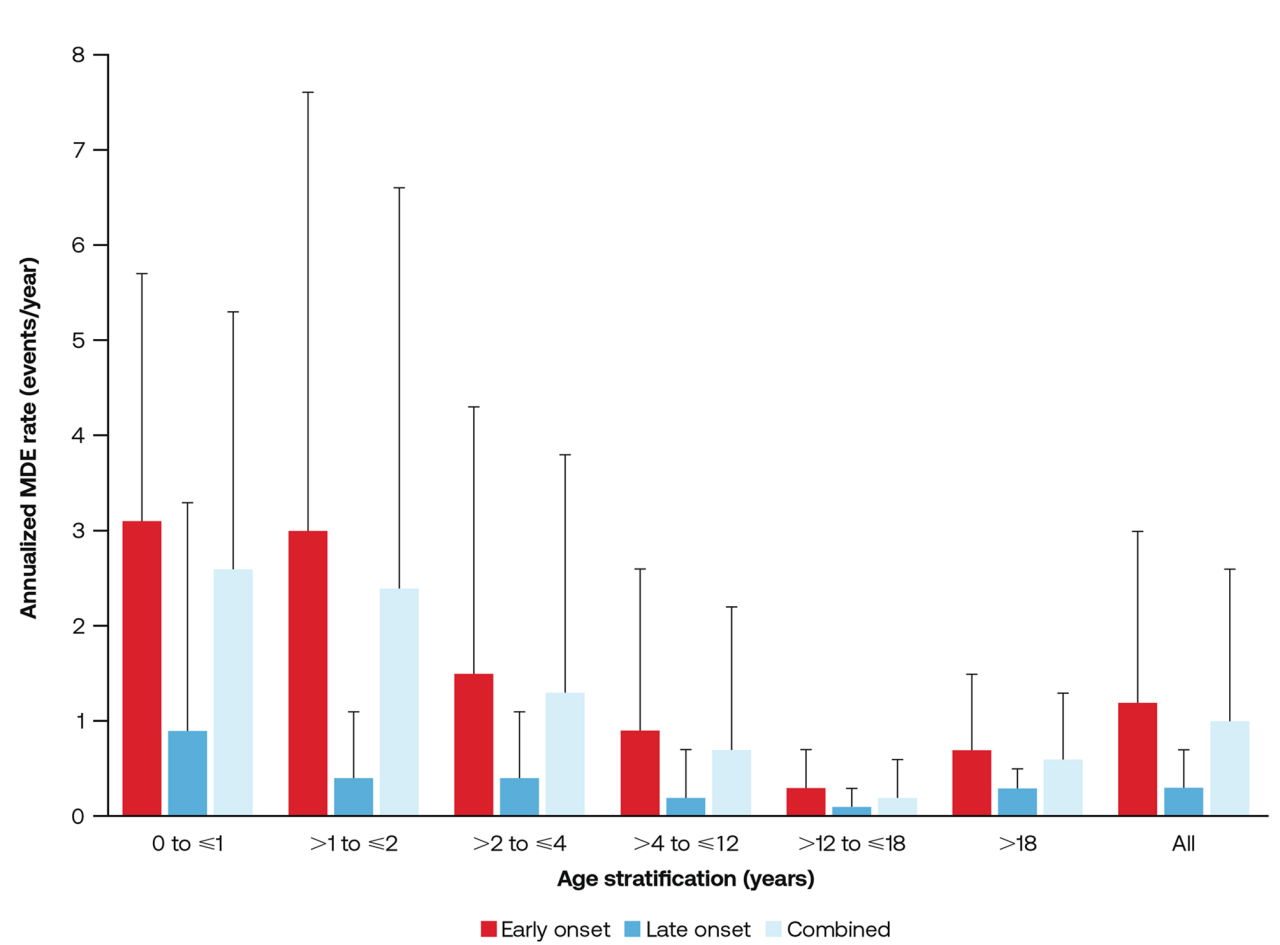
Mean (SD) annualized MDE rate for patients with PA by age at time of MDE and disease onset (events/year). MDE, metabolic decompensation event; PA, propionic acidemia; Sd, standard deviation

Overall, MDEs were reported in 98.0% (n/*N* = 49/50) of participants with PA, and the mean (standard deviation [SD]) annualized MDE frequency was 1.0 (1.6) (Fig. 2). The mean [SD] annualized MDE frequency was highest in participants ≤ 1 year (2.6 [2.7]) and lowest in participants > 12 years to ≥ 18 years (0.2 [0.4]) (Fig. 2). Overall, participants with early-onset PA had higher annualized MDE frequencies than participants with late-onset PA (1.2 [1.8] vs 0.3 [0.4]).

#### 3.3.1. Summary of MDEs by stratification group

Across all timepoints and all age stratification groups, participants consistently experienced vomiting and lethargy/decreased activity as the most common signs and symptoms of MDEs, and acidosis and hyperammonemia as the most common laboratory findings associated with MDEs. Participants who reported MDEs also consistently experienced MDE triggers of fever/infection or ‘other stressors’. Similar results were also observed across these timepoints when stratified by disease onset.

Overall, the proportion of MDEs that utilized hospitalization increased from 15.9% (n/*N* = 18/113 MDEs) at baseline to 100% (n/*N* = 13/13 MDEs) at month 24. Participants in the > 1 month to ≤ 2 years group had the highest proportion of MDEs utilizing hospitalization at baseline (n/*N* = 4/17 MDEs; 23.5%). At month 24, all 13 MDEs utilized hospitalization ( > 2 years to ≤ 12 years, n/*N* = 10/10 MDEs; > 18 years, 3/3 MDEs). At baseline, a similar proportion MDEs in participants with early- and late-onset PA utilized hospitalization (n/*N* = 16/100 MDEs, 16% vs n/*N* = 2/13 MDEs, 15.4%, respectively). At month 24, all MDEs in participants with early- and late-onset PA utilized hospitalization (n/*N* = 11/11 MDEs, 100% vs n/*N* = 2/2 MDEs, 100%, respectively).

#### Stratification by age at enrollment

#####  > 1 month to ≤ 2 years

**Fig. 3:**
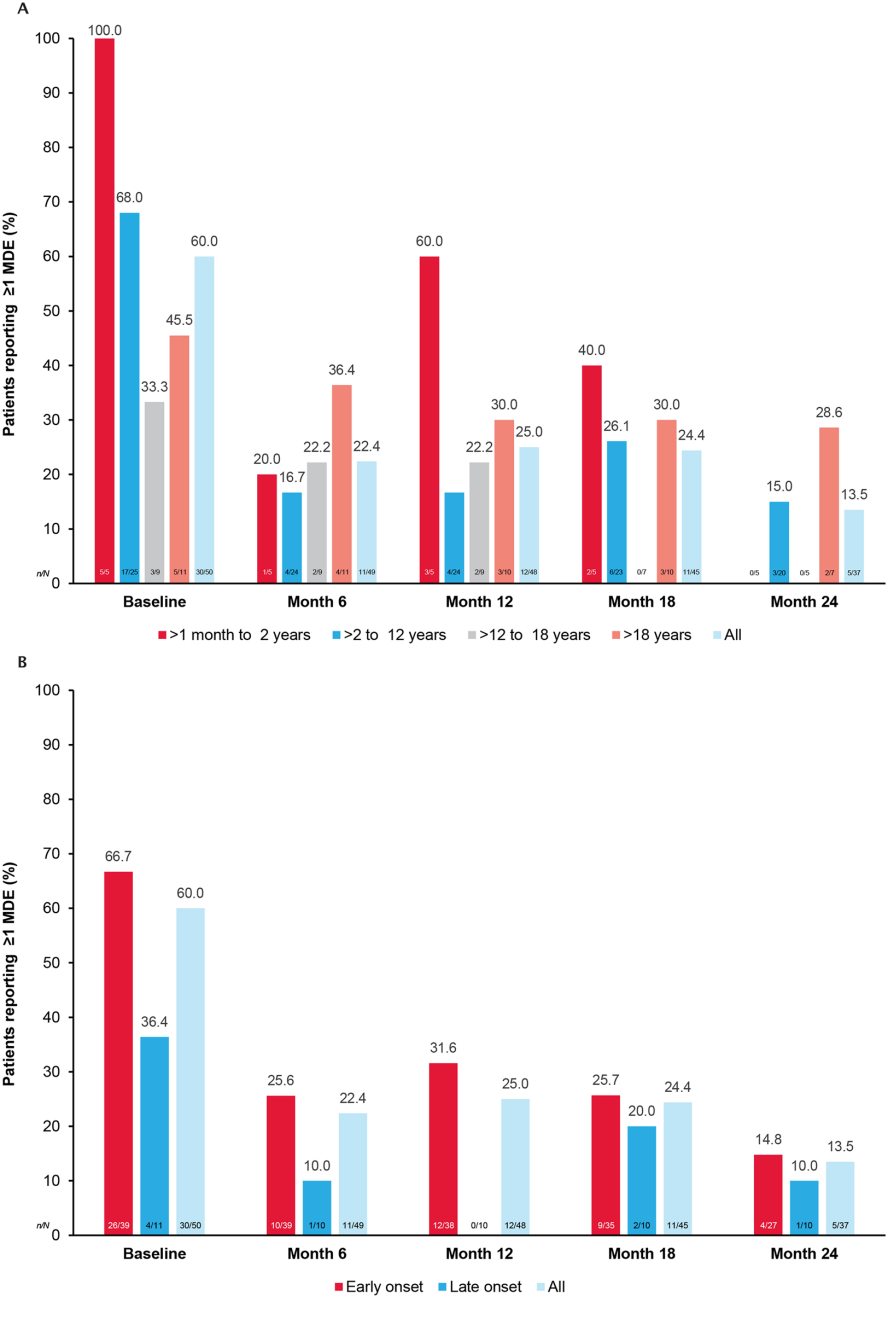
Patients with PA reporting ≥ 1 MDE at follow-ups over time, by (**A**) age and (**B**) disease onset. MDE, metabolic decompensation event; n/N, number of patients with ≥ 1 MDE event/number of patients at visit; PA, propionic acidemia. Baseline refers to data collected 365 days before to 60 days after enrollment)

The incidence of participants who experienced ≥ 1 MDE decreased from 100.0% (n/*N* = 5/5) at baseline to 40.0% (n/*N* = 2/5) at month 18 (no participants aged < 1 month experienced an MDE; Fig. 3 and Table S1). The mean (SD) annualized MDE rates for participants aged ≤ 1 year (*n* = 50) and > 1 year to ≤ 2 years (*n* = 50) were 2.6 (2.7) and 2.4 (4.2), respectively (Fig. 2). The most reported signs and symptoms of MDEs were vomiting, lethargy/decreased activity, and ‘other’, while the most reported laboratory findings were acidosis and hyperammonemia. Commonly reported MDE triggers were fever/infection and ‘other stressors’ (Table S2).

#####  > 2 years to ≤ 12 years

The incidence of participants who experienced ≥ 1 MDE decreased from 68.0% (n/*N* = 17/25) at baseline to 15.0% (n/*N* = 3/20) at Month 24 (Fig. 3 and Table S1). The mean (SD) annualized MDE rates for participants aged > 2 years to ≤ 4 years (*n* = 50) and > 4 years to ≤ 12 years (*n* = 45) were 1.3 (2.5) and 0.7 (1.5), respectively (Fig. 2). The most reported signs and symptoms of MDEs were vomiting, lethargy/decreased activity, and ‘other’, while the most reported laboratory finding was hyperammonemia. Commonly reported MDE triggers were fever/infection and ‘other stressors’ (Table S2).

#####  > 12 years to ≤ 18 years

The incidence of participants who experienced ≥ 1 MDE decreased from 33.3.% (n/*N* = 3/9) at baseline to 22.2% (n/*N* = 2/9) at month 12 (Fig. 3 and Table S1). The mean (SD) annualized MDE rates for participants aged > 12 years to ≤ 18 years (*n* = 21) was 0.2 (0.4) (Fig. 2). The most reported signs and symptoms of MDEs were lethargy/decreased activity and ‘other’, while the most reported laboratory findings were acidosis and hyperammonemia. Commonly reported MDE triggers were fever/infection and ‘other stressors’ (Table S2).

#####  > 18 years

The incidence of participants who experienced ≥ 1 MDE decreased from 45.5% (n/*N* = 5/11) at baseline to 28.6% (n/*N* = 2/7) at Month 24 (Fig. 3 and Table S1). The mean (SD) annualized MDE rates for participants aged > 18 years (*n* = 13) was 0.6 (0.7) (Fig. 2). The most reported signs and symptoms of MDEs were vomiting and lethargy/decreased activity, while the most reported laboratory finding was hyperammonemia. Commonly reported MDE triggers were ‘other stressors’ (Table 2).

#### Stratification by disease onset

##### Early-onset PA

The incidence of participants who experienced ≥ 1 MDE decreased from 66.7% (n/*N* = 26/39) at baseline to 14.8% (n/*N* = 4/27) at month 24 (Fig. 3 and Table S3). The mean (SD) annualized MDE rates for participants with early-onset PA (*n* = 39) was 1.2 (1.8) (Fig. 2). The most reported signs and symptoms of MDEs were vomiting, lethargy/decreased activity, and ‘other’, while the most reported laboratory findings were acidosis and hyperammonemia. Commonly reported MDE triggers were fever/infection and ‘other stressors’ (Table S4).

##### Late-onset PA

The incidence of participants who experienced ≥1 MDE decreased from 36.4% (n/*N* = 4/11) at baseline to 10.0% (n/*N* = 1/10) at month 24 (Fig. 3 and Table S3). The mean (SD) annualized MDE rates for participants with late-onset PA (*n* = 10) was 0.3 (0.4) (Fig. 2). The most reported signs and symptoms of MDEs at baseline were vomiting and ‘other, while the most frequently reported laboratory finding at baseline was hyperammonemia. The most reported MDE triggers at baseline were fever/infection and ‘other stressors’ (Table S4). No signs and symptoms of MDEs or MDE triggers were reported at other timepoints.

### MDEs in participants who underwent transplantation

Most participants with PA did not receive an organ transplant, while 16% (n/*N* = 8/50) received a liver transplant. Participants who did not receive a transplant had a higher mean (SD) annualized MDE rate compared to participants who received a transplant (1.1 [1.7] vs 0.8 [1.3]). Further, participants who received a liver transplant showed a decrease in mean (SD) annualized MDE rates from 1.0 (2.3) pre-transplant to 0.5 (0.8) post-transplant.

### Baseline biomarker concentrations

**Fig. 4:**
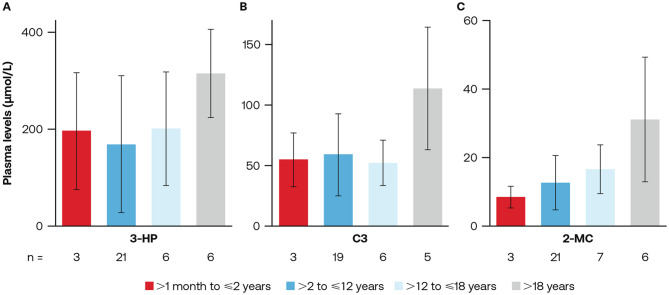
Plasma mean levels of (**A**) 3-HP, (**B**) C3, and (**C**) 2-MC in patients with PA by age at baseline^a^. 2-MC, 2-methylcitrate; 3-HP, 3-hydroxypropionic acid; C3, propionylcarnitine; PA, propionic acidemia. ^a^Due to small sample sizes and high variability during follow-up, only baseline biomarkers are presented

There were no significant trends observed between baseline biomarker concentrations and age for 3-HP, C3, and 2-MC (Fig. 4 A-C).

### Correlation between biomarkers and MDEs

Utilizing the GAMM framework, it was observed that a 50% lower plasma 3-HP concentration was associated with a 30% lower frequency of MDEs (RR, 0.7; 95% CI, 0.6, 0.9; *p* < 0.01). These results were consistent with the negative binomial regression methodology, which demonstrated that a 50% lower plasma 3-HP concentration was associated with a 20% lower frequency of MDE, though these results were not statistically significant (RR, 0.8; 95% CI, 0.5, 1.2; *p* = 0.25).

When observing the RR of MDEs in relation to other biomarker concentrations, a 50% lower plasma C3 concentrations was associated with a 40% lower RR of MDEs using binomial regression (RR, 0.6; 95% CI, 0.4, 0.9; *p* = 0.017). However, a 50% lower plasma 2-MC concentration was not associated with a significantly lower MDE frequency using binomial regression (RR, 1.0; 95% CI, 0.7, 1.3; *p* = 0.84).

## Discussion

Heterogeneity of disease presentation and clinical symptomatology in the setting of ultra-rare metabolic disorders such as PA can complicate the establishment of clinical trials and present challenges for advancing diagnostic and treatment strategies. This has been further complicated by a lack of comprehensive natural history studies for PA. The present study explored disease defining endpoints of PA and examined potential links between clinical events and biomarkers, with the aim of improving the general understanding of PA disease characteristics. All but one participant experienced an MDE throughout the study, with annualized rates of MDEs highest in participants aged ≤ 2 years (≤ 1 year, 2.6; > 1 year to ≤ 2 years, 2.4) and lowest in participants aged ≥ 12 years ( > 12 years to ≤ 18 years, 0.2; > 18 years, 0.6). Seizures were a frequently reported disease complication at baseline, regardless of age group; however, participants with early-onset PA were more likely to have seizures compared with late-onset PA. Participants aged > 2 years to ≤ 12 years, and those with early-onset PA were more likely to experience pancreatitis than other participant groups. Further, participants ≤ 12 years old and those with early-onset PA were more likely to experience signs and symptoms of MDEs longer term (18–24 months) than participants > 12 years old or with late-onset PA, respectively. While there was no reduced risk of MDEs associated with lower levels of 2-MC, lower plasma 3-HP and C3 concentrations were associated with reduced risks of MDEs.

When examining baseline disease complications and symptoms, seizures were the most common complication among all participants, regardless of age or timing of disease onset, with nearly half of all participants reporting them. These results are aligned with previous studies showing that seizures are one of the most common acute neurological presentations in PA [1, 7, 13]. Pancreatitis was the second most common complication and was observed in approximately one-fifth of all participants with PA in this study. While pancreatitis has been observed less frequently than neurological complications [5], the frequencies reported here were similar to those observed in previous studies [10].

We observed a persistence of MDEs over time, particularly among younger participants (≤ 2 years of age). Here we report that participants with early-onset PA had more frequent MDEs than participants with late-onset PA. Previous reports have shown MDE rates in participants with PA being highest during the first three years of life and, although spikes may occur later in life, frequency typically decreases with age [11, 14]. This decrease could be related to multiple factors, including low-protein dietary adherence, improved metabolic control through medical supplements, decreased frequency of intercurrent illnesses which trigger MCEs and, improved metabolic control after liver transplantation [15, 16]. It is also possible that adaptations of metabolic pathways might improve with age as the body has less metabolic demands. Poor metabolic reserves have been observed to cause increased ketoacidosis and idiopathic ketotic hypoglycemia in children approximately aged 3–5 years; however, these conditions typically improve with age, and are often resolved between 8 and 9 years of age [17, 18]. While the reporting of signs and symptoms of MDEs can provide additional natural history information for participants with PA, challenges surrounding participant population, health care access and systems, study frequency, and study duration exist which may complicate findings [11, 19].

In this study, analysis of biomarkers showed that lower plasma 3-HP and C3 concentrations were associated with a statistically significant lower risk of MDEs, 30% for 3-HP and 40% for C3. The significance observed with 3-HP aligns with previous literature where it has been identified as one of the primary metabolites that accumulates in participants with PA due to decreased enzymatic activity [7]. Concentrations of 3-HP correlate with periods of metabolic instability; for example, 3-HP concentrations may be up to three times higher in participants with MDEs than those without MDEs [20]. Additional evidence has shown 3-HP concentrations to be significantly reduced following liver transplantation [20]. The relationship between reduced 3-HP concentrations and reduced risk of MDEs observed in this study, combined with the previous evidence, provides further support for 3-HP as a potential biomarker of disease severity that can help to better characterize the impacts of disease modifying interventions in clinical trials for participants with PA. Identification of biomarkers is an important component of natural history studies, as they may help determine correlations with clinical endpoints to guide future diagnostic and treatment strategies. However, plasma concentrations of these biomarkers are highly dynamic and, in some cases, can be difficult to link to an individual participant’s clinical status and may not represent their overall severity [4]. While the presence of certain biomarkers may contribute to the evolution of metabolic crises in participants with PA, further investigation is required to determine whether they are sufficient to reflect the risk of MDE in future clinical trials, to replicate and validate these findings and to assist in finding novel treatment options for participants with PA. With regards to the present study, it is important to note small sample sizes and high variability within stratification groups limited the analysis to baseline readings.

This study showed that participants who received liver transplants had lower annualized MDE rates than participants of the same age who had not received transplants. Transplantation has been associated with improved metabolic stability in participants with organic acidemias and is thought to be related to the transplanted liver providing enzyme activity [1, 8]. Additional larger follow-up studies are needed to determine whether there are correlations between transplantation and changes in clinical outcomes in participants with PA.

Future studies into the natural history of PA should focus on long-term and participant–observer clinical outcomes. An expanded investigation into the impact of biomarkers such as 3-HP and C3 is also required to determine their importance in PA clinical trials. This study provides comprehensive and reliable clinical and biomarker data to further characterize the natural history of participants with PA. The study design allowed for analysis of outcomes by age and disease onset, along with transplantation status, for a more comprehensive dataset. Ultimately, data collected in this study may provide insights into outcomes of interest that could be used to develop treatments for PA in future trials.

Several limitations were present in this study. Recall bias and channeling bias may have been present, and results may not be generalizable to the overall participant population. The definition of MDEs was not independently adjudicated, and there could have been differences in interpretation and application of definitions across sites and between clinicians. MDE rates may have been artificially adjusted by participants attempting to treat MDEs at home, or by carefully managing diet based on early signs/symptoms. The definitions of early- and late-onset PA used in this study (first MDE before or after three months of age) differed from previous studies, where definitions vary (for example, during versus after the neonatal period [ie, 28 days old], or during first year of life versus after first year of life) [6, 21]. The dataset was not powered for binomial regression analyses. The study required participants to survive at least the first month of life, therefore the burden/severity of disease may be underestimated and may have caused survival bias. Conversely, participants who were less severely affected or could manage their disease as they aged may also not have participated fully or had fewer clinic visits. Further, long-term follow-ups were limited in the study. Finally, this study was conducted in part during the COVID-19 pandemic where quarantine/homeschooling/working from home led to potentially lower rates of MDE’s, with reduced risk exposure to triggers such as intercurrent infections other than COVID-19 infection.

## Conclusion

Advancement in the diagnosis and treatment of rare metabolic disorders like PA require additional natural history characterization and further understanding of the relationship between clinical events and biomarkers. This study evaluated and described the natural history of participants with PA and examined relationships between clinical events and biomarker concentrations. Younger participants (≤ 2 years) and those diagnosed with early-onset PA ( > 75% of the study population) appeared to have the greatest annualized MDE frequencies over longer periods of follow-up (compared with older participants and those with late-onset PA). Lower levels of 3-HP and C3 concentrations showed correlations with reduced risk of MDEs. To our knowledge, this is the first and largest study of its kind to examine longitudinal MDE rates across multiple stratifications (age and disease onset) and their relationship to PA-specific biomarkers. These results may help to expand knowledge of PA management and may provide additional insights into the development of future clinical trials.

## Electronic supplementary material.

Below is the link to the electronic supplementary material.

Supplementary material 1

## Abbreviations

2-MC: 2-methylcitrate
3-HP: 3-hydroxypropionic acid
C3: Propionlycarnitine
CoA: Coenzyme A
DBS: Dried Blood Spot
GAMM: Generalized additive mixed modeling
NBS: Newborn screening
MDE: Metabolic decompensation event
PA: Propionic acidemia
SD: Standard deviation

## Declarations

### Data availability

Access to participant-level data presented in this article and supporting clinical documents with external researchers who provide methodologically sound scientific proposals will be available upon reasonable request for products or indications that have been approved by regulators in the relevant markets and subject to review. Such requests can be made to: Moderna Inc., 325 Binney St., Cambridge, MA 02142 USA or at data_sharing@modernatx.com. A materials transfer and/or data access agreement with the sponsor will be required for accessing shared data. All other relevant data are presented in the paper. The protocol is available online at ClinicalTrials.gov: NCT03484767; registered on 2 April 2018.
