# Supplementary material for: Characterizing the frequency of clinical events and assessing biomarkers in propionic acidemia: a natural history study
Source: Orphanet J Rare Dis. 2026 Mar 9;21:152. doi: 10.1186/s13023-026-04251-3 (PMC13085267; doi:10.1186/s13023-026-04251-3)
Supplement: Supplementary file 1 — Supplementary material 1 [file 13023_2026_4251_MOESM1_ESM.docx]

- **Supplementary Appendix**
- **Table S1.**
- Patients reporting ≥1 MDE, mean annualized MDE rates, and mean MDE durations for patients with PA stratified by age at enrollment, over time.

|  | Baseline | Month 6 | Month 12 | Month 18 | Month 24 |
| --- | --- | --- | --- | --- | --- |
| >1 month to ≤2 years | | | | | |
| Patients reporting ≥1 MDE, % (n/N) | 100.0 (5/5) | 20.0 (1/5) | 60.0 (3/5) | 40.0 (2/5) | - |
| Mean annualized MDE rate (SD) | 2.9 (1.2) | 1.2 (2.7) | 2.0 (2.0) | 2.0 (2.8) | - |
| Mean annualized MDE duration, days (SD) | 7.8 (3.8) | 3.3 (0.6) | 9.3 (9.3) | 4.0 (1.4) | - |
| >2 to ≤12 years | | | | | |
| Patients reporting ≥1 MDE, % (n/N) | 68.0 (17/25) | 16.7 (4/24) | 16.7 (4/24) | 26.1 (6/23) | 15.0 (3/20) |
| Mean annualized MDE rate (SD) | 2.4 (2.3) | 1.3 (4.0) | 1.1 (3.2) | 1.1 (2.0) | 1.0 (2.8) |
| Mean annualized MDE duration, days (SD) | 4.3 (2.4) | 3.4 (0.7) | 3.0 (1.0) | 3.6 (1.6) | 3.3 (0.7) |
| >12 to ≤18 years | | | | | |
| Patients reporting ≥1 MDE, % (n/N) | 33.3 (3/9) | 22.2 (2/9) | 22.2 (2/9) | - | - |
| Mean annualized MDE rate (SD) | 0.5 (0.9) | 0.5 (0.9) | 2.1 (3.9) | - | - |
| Mean annualized MDE duration, days (SD) | 8.0 (8.5) | 12.5 (10.6) | 6.5 (0.7) | - | - |
| >18 years | | | | | |
| Patients reporting ≥1 MDE, % (n/N) | 45.5 (5/11) | 36.4 (4/11) | 30.0 (3/10) | 30.0 (3/10) | 28.6 (2/7) |
| Mean annualized MDE rate (SD) | 1.6 (3.2) | 1.5 (2.5) | 0.6 (1.0) | 1.0 (1.9) | 0.9 (1.6) |
| Mean annualized MDE duration, days (SD) | 5.9 (4.6) | 26.9 (54.3) | 7.3 (4.2) | 6.2 (2.3) | 18.0 (13.5) |

- n/N = number of patients reporting ≥1 MDE/number of patients with data available. No data to report for months 30 and 36. ‘-’ indicates that either <5 patients were present at the visit or no patients displayed ≥1 MDE and data was not collected. MDE, metabolic decompensation event; PA, propionic acidemia; Sd, standard deviation.
- **Table S2.**
- Signs, symptoms, and/or clinical manifestations of MDEs and common MDE triggers for patients with PA stratified by age at enrollment, over time.

|  | | Baseline | | Month 6 | | Month 12 | | Month 18 | Month 24 |
| --- | --- | --- | --- | --- | --- | --- | --- | --- | --- |
| >1 month to ≤2 years | | | | | | | | | |
| Signs and symptoms of MDEs^a^ | | | | | | | | | |
| Vomiting, % (n/N) | 60.0 (3/5) | | 100.0 (1/1) | | 33.3 (1/3) | | 100.0 (2/2) | | - |
| Acidosis, % (n/N) | 40.0 (2/5) | | 100.0 (1/1) | | 66.7.0 (2/3) | | 100.0 (2/2) | | - |
| Hyperammonemia, % (n/N) | 80.0 (4/5) | | 100.0 (1/1) | | 33.3 (1/3) | | 100.0 (2/2) | | - |
| Food refusal, % (n/N) | 40.0 (2/5) | | 100.0 (1/1) | | N/A | | N/A | | - |
| Impaired consciousness, % (n/N) | 40.0 (2/5) | | N/A | | 33.3 (1/3) | | 50.0 (1/2) | | - |
| Lethargy/decreased activity, % (n/N) | 60.0 (3/5) | | N/A | | 33.3 (1/3) | | 50.0 (1/2) | | - |
| Generalized change in behavior, % (n/N) | 40.0 (2/5) | | N/A | | 33.3 (1/3) | | 50.0 (1/2) | | - |
| ‘Other’, % (n/N) | 60.0 (3/5) | | N/A | | 33.3 (1/3) | | 50.0 (1/2) | | - |
| Common MDE triggers^b^ | | | | | | | | | |
| Fever/infection, % (n/N) | 100.0 (4/4) | | 100.0 (1/1) | | 100.0 (2/2) | | 50.0 (1/2) | | - |
| Surgery, % (n/N) | 25.0 (1/4) | | N/A | | 50.0 (1/2) | | N/A | | - |
| Inappropriate sick day treatment protocol implementation, % (n/N) | N/A | | 100.0 (1/1) | | N/A | | N/A | | - |
| Insufficient calories, % (n/N) | 25.0 (1/4) | | N/A | | N/A | | N/A | | - |
| Vaccination, % (n/N) | N/A | | N/A | | N/A | | N/A | | - |
| Constipation, % (n/N) | N/A | | 100.0 (1/1) | | N/A | | N/A | | - |
| ‘Other stressors’, % (n/N) | 75.0 (3/4) | | 100.0 (1/1) | | 50.0 (1/2) | | 100.0 (2/2) | | - |
| >2 to ≤12 years | | | | | | | | | |
| Signs and symptoms of MDEs^a^ | | | | | | | | | |
| Vomiting, % (n/N) | | 88.2 (15/17) | | 75.0 (3/4) | | 75.0 (3/4) | | 83.3 (5/6) | 100.0 (3/3) |
| Acidosis, % (n/N) | | N/A | | N/A | | 25.0 (1/4) | | N/A | 33.3 (1/3) |
| Hyperammonemia, % (n/N) | | 64.7 (11/17) | | 50.0 (2/4) | | 75.0 (3/4) | | 83.3 (5/6) | 100.0 (3/3) |
| Food refusal, % (n/N) | | N/A | | N/A | | N/A | | N/A | 33.3 (1/3) |
| Impaired consciousness, % (n/N) | | N/A | | N/A | | N/A | | N/A | 33.3 (1/3) |
| Lethargy/decreased activity, % (n/N) | | 58.8 (10/17) | | 50.0 (2/4) | | 50.0 (2/4) | | N/A | 100.0 (3/3) |
| Generalized change in behavior, % (n/N) | | N/A | | N/A | | 25.0 (1/4) | | N/A | 33.3 (1/3) |
| ‘Other’, % (n/N) | | 52.9 (9/17) | | 50.0 (2/4) | | 75.0 (3/4) | | 66.7 (4/6) | 66.7 (2/3) |
| Common MDE triggers^b^ | | | | | | | | | |
| Fever/infection, % (n/N) | | 82.4 (14/17) | | 33.3 (1/3) | | 50.0 (2/4) | | 40.0 (2/5) | 100.0 (3/3) |
| Surgery, % (n/N) | | N/A | | N/A | | N/A | | N/A | N/A |
| Inappropriate sick day treatment protocol implementation, % (n/N) | | N/A | | N/A | | N/A | | N/A | N/A |
| Insufficient calories, % (n/N) | | 35.3 (6/17) | | 66.7 (2/3) | | 50.0 (2/4) | | 40.0 (2/5) | 33.3 (1/3) |
| Vaccination, % (n/N) | | N/A | | N/A | | 25.0 (1/4) | | N/A | N/A |
| Constipation, % (n/N) | | N/A | | N/A | | 25.0 (1/4) | | N/A | N/A |
| ‘Other stressors’, % (n/N) | | 88.2 (15/17) | | 100.0 (3/3) | | 50.0 (2/4) | | 80.0 (4/5) | 66.7 (2/3) |
| >12 to ≤18 years | | | | | | | | | |
| Signs and symptoms of MDEs^a^ | | | | | | | | | |
| Vomiting, % (n/N) | | 100.0 (3/3) | | N/A | | N/A | | - | - |
| Acidosis, % (n/N) | | 33.3 (1/3) | | 50.0 (1/2) | | 50.0 (1/2) | | - | - |
| Hyperammonemia, % (n/N) | | 66.7 (2/3) | | 100.0 (2/2) | | N/A | | - |  |
| Food refusal, % (n/N) | | N/A | | N/A | | 50.0 (1/2) | | - | - |
| Impaired consciousness, % (n/N) | | N/A | | 50.0 (1/2) | | N/A | | - | - |
| Lethargy/decreased activity, % (n/N) | | 33.3 (2/3) | | 50.0 (1/2) | | N/A | | - | - |
| Generalized change in behavior, % (n/N) | | N/A | | N/A | | N/A | | - | - |
| ‘Other’, % (n/N) | | 66.7 (2/3) | | N/A | | 50.0 (1/2) | | - | - |
| Common MDE triggers^b^ | | | | | | | | | |
| Fever/infection, % (n/N) | | 66.7 (2/3) | | 100.0 (2/2) | | 100.0 (2/2) | | - | - |
| Surgery, % (n/N) | | N/A | | N/A | | N/A | | - | - |
| Inappropriate sick day treatment protocol implementation, % (n/N) | | N/A | | N/A | | N/A | |  | - |
| Insufficient calories, % (n/N) | | N/A | | N/A | | N/A | | - | - |
| Vaccination, % (n/N) | | N/A | | N/A | | N/A | | - | - |
| Constipation, % (n/N) | | N/A | | N/A | | N/A | | - | - |
| ‘Other stressors’, % (n/N) | | 33.3 (1/3) | | 50.0 (1/2) | | N/A | | - | - |
| >18 years | | | | | | | | | |
| Signs and symptoms of MDEs^a^ | | | | | | | | | |
| Vomiting, % (n/N) | | 80.0 (4/5) | | 75.0 (3/4) | | 33.3 (1/3) | | 33.3 (1/3) | N/A |
| Acidosis, % (n/N) | | 40.0 (2/5) | | 50.0 (2/4) | | N/A | | 66.7 (2/3) | N/A |
| Hyperammonemia, % (n/N) | | N/A | | 75.0 (3/4) | | 66.7 (2/3) | | 66.7 (2/3) | 50.0 (1/2) |
| Food refusal, % (n/N) | | N/A | | 25.0 (1/4) | | N/A | | N/A | N/A |
| Impaired consciousness, % (n/N) | | 40.0 (2/5) | | 50.0 (2/4) | | N/A | | 66.7 (2/3) | N/A |
| Lethargy/decreased activity, % (n/N) | | 40.0 (2/5) | | 75.0 (3/4) | | 33.3 (1/3) | | 66.7 (2/3) | N/A |
| Generalized change in behavior, % (n/N) | | N/A | | 75.0 (3/4) | | 33.3 (1/3) | | N/A | N/A |
| ‘Other’, % (n/N) | | 60.0 (3/5) | | 25.0 (1/4) | | 33.3 (1/3) | | N/A | N/A |
| Common MDE triggers^b^ | | | | | | | | | |
| Fever/infection, % (n/N) | | 75.0 (3/4) | | N/A | | N/A | | N/A | N/A |
| Surgery, % (n/N) | | N/A | | N/A | | N/A | | N/A | N/A |
| Inappropriate sick day treatment protocol implementation, % (n/N) | | N/A | | N/A | | N/A | | N/A | N/A |
| Insufficient calories, % (n/N) | | N/A | | N/A | | N/A | | N/A | N/A |
| Vaccination, % (n/N) | | N/A | | N/A | | N/A | | N/A | N/A |
| Constipation, % (n/N) | | N/A | | N/A | | N/A | | 50.0 (1/2) | N/A |
| ‘Other stressors’, % (n/N) | | 75.0 (3/4) | | 100.0 (4/4) | | 100.0 (3/3) | | 50.0 (1/2) | N/A |

- ^a^n/N = number of patients with sign or symptom/number of patients reporting ≥1 MDE.
- ^b^n/N = number of patients with trigger/number of patients reporting ≥1 MDE trigger.
- No data to report for Month 30s and 36. ‘-‘ indicates that no data was available to record; ‘N/A’ indicates that <25% of patients displayed specific signs, symptoms, and/or clinical manifestations of MDEs or common MDE triggers. MDE, metabolic decompensation event; PA, propionic acidemia.
- **Table S3.**
- Patients reporting ≥1 MDE, mean annualized MDE rates, and mean MDE durations for patients with PA stratified by disease onset, over time.

|  | Baseline | Month 6 | Month 12 | Month 18 | Month 24 |
| --- | --- | --- | --- | --- | --- |
| Early-onset PA | | | | | |
| Patients reporting ≥1 MDE, % (n/N) | 66.7 (26/39) | 25.6 (10/39) | 31.6 (12/38) | 25.7 (9/35) | 14.8 (4/27) |
| Mean annualized MDE rate (SD) | 2.2 (2.5) | 1.4 (3.5) | 1.6 (3.2) | 1.1 (2.1) | 0.9 (2.5) |
| Mean annualized MDE duration, days (SD) | 5.4 (3.8) | 10.8 (29.7) | 5.1 (4.6) | 4.2 (1.7) | 6.2 (9.1) |
| Late-onset PA | | | | | |
| Patients reporting ≥1 MDE, % (n/N) | 36.4 (4/11) | 10.0 (1/10) | - | 20.0 (2/10) | 10.0 (1/10) |
| Mean annualized MDE rate (SD) | 1.0 (1.7) | 0.2 (0.7) | - | 0.7 (1.4) | 0.4 (1.3) |
| Mean annualized MDE duration, days (SD) | 4.6 (3.0) | - | - | 5.5 (5.0) | 11.0 (8.5) |

- n/N = number of patients reporting ≥1 MDE/number of patients with data available. No data to report for Months 30 and 36. ‘-’ indicates that either <5 patients were present at the visit or no patients displayed ≥1 MDE and data was not collected. MDE, metabolic decompensation event; PA, propionic acidemia; SD, standard deviation.
- **Table S4.**
- Signs, symptoms, and or clinical manifestations of MDEs and common MDE triggers for patients with PA stratified by disease onset, over time.

|  | Baseline | Month 6 | Month 12 | Month 18 | Month 24 |
| --- | --- | --- | --- | --- | --- |
| Early-onset PA | | | | | |
| Signs and symptoms of MDEs^a^ | | | | | |
| Vomiting, % (n/N) | 84.6 (22/26) | 70.0 (7/10) | 41.7 (5/12) | 88.9 (8/9) | 75.0 (3/4) |
| Acidosis, % (n/N) | 34.6 (9/26) | 40.0 (4/10) | 33.3 (4/12) | 55.6 (5/9) | 25.0 (1/4) |
| Hyperammonemia, % (n/N) | 65.4 (17/26) | 80.0 (8/10) | 50.0 (6/12) | 100.0 (9/9) | 100.0 (4/4) |
| Food refusal, % (n/N) | N/A | N/A | N/A | N/A | 25.0 (1/4) |
| Impaired consciousness, % (n/N) | N/A | 30.0 (3/10) | N/A | 33.3 (3/9) | 25.0 (1/4) |
| Lethargy/decreased activity, % (n/N) | 61.5 (16/26) | 60.0 (6/10) | 33.3 (4/12) | 33.3 (3/9) | 75.0 (3/4) |
| Generalized change in behavior, % (n/N) | N/A | 30.0 (3/10) | 25.0 (3/12) | N/A | 25.0 (1/4) |
| ‘Other’, % (n/N) | 53.8 (14/26) | 30.0 (3/10) | 50.0 (6/12) | 55.6 (5/9) | 50.0 (2/4) |
| Common MDE triggers^b^ | | | | | |
| Fever/infection, % (n/N) | 87.5 (21/24) | 40.0 (4/10) | 54.5 (6/11) | 33.3 (3/9) | 100.0 (3/3) |
| Surgery, % (n/N) | N/A | N/A | N/A | N/A | N/A |
| Inappropriate sick day treatment protocol implementation, % (n/N) | N/A | N/A | N/A | N/A | N/A |
| Insufficient calories, % (n/N) | 25.0 (6/24) | N/A | N/A | N/A | 33.3 (1/3) |
| Vaccination, % (n/N) | N/A | N/A | N/A | N/A | N/A |
| Constipation, % n/N) | N/A | N/A | N/A | N/A | N/A |
| ‘Other stressors’, % (n/N) | 79.2 (19/24) | 90.0 (9/10) | 54.5 (6/11) | 77.8 (7/9) | 66.7 (2/3) |
| Late-onset PA | | | | | |
| Signs and symptoms of MDEs^a^ | | | | | |
| Vomiting, % (n/N) | 75.0 (3/4) | - | - | - | - |
| Acidosis, % (n/N) | N/A | - | - | - | - |
| Hyperammonemia, % (n/N) | 25.0 (1/4) | - | - | - | - |
| Food refusal, % (n/N) | N/A | - | - | - | - |
| Impaired consciousness, % (n/N) | 50.0 (2/4) | - |  |  |  |
| Lethargy/decreased activity, % (n/N) | 25.0 (1/4) | - | - | - | - |
| Generalized change in behavior, % (n/N) | 25.0 (1/4) | - |  |  |  |
| ‘Other’, % (n/N) | 75.0 (3/4) | - | - | - | - |
| Common MDE triggers^b^ | | | | | |
| Fever/infection, % (n/N) | 50.0 (2/4) | - | - | - | - |
| Surgery, % (n/N) | N/A | - | - | - | - |
| Inappropriate sick day treatment protocol implementation, % (n/N) | N/A | - | - | - | - |
| Insufficient calories, % (n/N) | 25.0 (1/4) | - | - | - | - |
| Vaccination, % (n/N) | N/A | - | - | - | - |
| Constipation, % (n/N) | 25.0 (1/4) | - | - | - | - |
| ‘Other stressors’, % (n/N) | 75.0 (3/4) | - | - | - | - |

- ^a^n/N = number of patients with sign or symptom/number of patients reporting ≥1 MDE.
- ^b^n/N = number of patients with trigger/number of patients reporting ≥1 MDE trigger.
- No data to report for months 30 and 36. ‘-‘ indicates that no data was available to record; ‘N/A’ indicates that <25% of patients displayed specific signs and symptoms of MDEs or common MDE triggers. MDE, metabolic decompensation event; PA, propionic acidemia.
